# APOE ε4 gene dose drives a canonical and predictable topography of amyloid deposition

**DOI:** 10.1002/alz.71935

**Published:** 2026-10-29

**Authors:** Augusto J. Mendes, Cecilia Boccalini, Federica Ribaldi, Michela Pievani, Débora Peretti, Valentina Garibotto, Andrea Chincarini, Giovanni B. Frisoni, for the Alzheimer's Disease Neuroimaging Initiative

**Affiliations:** ^1^ Laboratory of Neuroimaging of Aging (LANVIE) University of Geneva Geneva Switzerland; ^2^ Geneva Memory Center Department of Rehabilitation and Geriatrics Geneva University Hospitals Geneva Switzerland; ^3^ Laboratory of Neuroimaging and Innovative Molecular Tracers (NIMTlab), Geneva University Neurocenter and Faculty of Medicine University of Geneva Geneva Switzerland; ^4^ Laboratory of Alzheimer's Neuroimaging and Epidemiology (LANE) IRCCS Istituto Centro San Giovanni di Dio Fatebenefratelli Brescia Italy; ^5^ Division of Nuclear Medicine and Molecular Imaging Geneva University Hospitals Geneva Switzerland; ^6^ CIBM Center for Biomedical Imaging Geneva Switzerland; ^7^ Genoa section National Institute of Nuclear Physics (INFN) Rome Genoa Italy

**Keywords:** Alzheimer, amyloid, APOE ε4, genetic, topography

## Abstract

**INTRODUCTION:**

Apolipoprotein E ε4 (APOE ε4) carriership is associated with higher amyloid burden in Alzheimer's disease (AD), but whether it constrains the spatial predictability of amyloid deposition at the individual level remains unknown.

**METHODS:**

We analyzed voxel‐wise amyloid positron emission tomography data from 781 amyloid‐positive individuals across two independent cohorts (Alzheimer's Disease Neuroimaging Initiative and Geneva Memory Center), spanning the full clinical continuum. Individual topographic similarity to canonical AD amyloid maps and within‐group spatial homogeneity were assessed via voxel‐wise correlations and linear regression adjusted for age, sex, and global amyloid burden.

**RESULTS:**

APOE ε4 carriers exhibited amyloid distributions more aligned with canonical AD topographies than non‐carriers (*p *< 0.001), with a clear gene‐dose effect on within‐group spatial homogeneity: Homozygotes showed the highest inter‐subject consistency, followed by heterozygotes and non‐carriers. Effects were robust across tracers, clinical stages, and cohorts.

**DISCUSSION:**

APOE ε4 gene dose constrains the spatial predictability of amyloid accumulation, informing genotype‐stratified approaches in anti‐amyloid trials.

## Highlights

- APOE ε4 gene dose increases the spatial predictability of amyloid deposition.
- APOE ε4 carriers show greater alignment with canonical AD amyloid topographies.
- Homozygotes exhibit the highest inter‐individual spatial homogeneity of amyloid.
- Effects were replicated in both cohorts and independent of global amyloid burden.
- Findings support APOE ε4‐stratified strategies in anti‐amyloid trials.

## 1 INTRODUCTION

Why amyloid deposition follows predictable spatial trajectories in some individuals but not others is a fundamental, unresolved question in Alzheimer's disease (AD) research, with direct consequences for the emerging era of anti‐amyloid therapies. The classical amyloid cascade hypothesis posits a largely deterministic sequence of events whereby amyloid deposition triggers tau aggregation, followed by neurodegeneration and, ultimately, progressive cognitive decline.^1^ However, growing evidence points to substantial heterogeneity in the onset, regional distribution, and rate of progression of AD pathology, as well as in its clinical manifestations, suggesting that this process may vary substantially across individuals.^2^, ^3^

To address this complexity, several variants of the deterministic version of the amyloid cascade hypothesis have been proposed. One example is the probabilistic model of AD, which reinterprets the cascade by integrating both genetic and stochastic factors into the sequence of events leading to cognitive impairment and progression.^4^ Within this framework, apolipoprotein E ε4 allele (APOE ε4) carriership, the strongest common genetic risk factor for sporadic AD,^5^ is a key biological modifier of the cascade's penetrance. The model proposes that the degree of determinism in the amyloid‐to‐tau‐to‐neurodegeneration sequence varies according to the strength of the genetic factors. Accordingly, three major forms of AD can be distinguished: autosomal dominant AD (ADAD), APOE ε4‐related sporadic AD, and APOE ε4‐unrelated sporadic AD. These categories represent a continuum of decreasing pathophysiological determinism and increasing influence of stochastic factors, such as lifestyle behaviors and exposures.^4^ In ADAD, due to the presence of a deterministic mutation, amyloid accumulation, and subsequent pathological events in the AD cascade occur inevitably and follow a relatively predictable trajectory. In contrast, in APOE ε4‐unrelated sporadic AD, amyloid deposition and the downstream pathological processes are increasingly heterogeneous and less predictable, reflecting a greater influence of non‐genetic and modifiable factors.^4^ Furthermore, a growing body of evidence has emphasized the gene‐dose effect of APOE ε4, as homozygosity confers a markedly higher penetrance and earlier onset of pathology.^6^, ^7^ This has raised the question of whether APOE ε4 homozygosity constitutes a genetic form of AD.^8^, ^9^, ^10^, ^11^

Despite previous evidence that APOE ε4 modulates global amyloid burden^12^ or regional differences between APOE ε4 genotypes,^13^ it has not been demonstrated whether APOE ε4 constrains the topographic predictability of amyloid deposition at the individual level. Prior studies have characterized regional differences in amyloid uptake between APOE ε4 genotype groups, but group‐level comparisons do not capture the degree to which each individual's amyloid positron emission tomography (PET) pattern resembles a canonical AD spatial template, nor do they assess within‐genotype spatial consistency across subjects. These are distinct and complementary dimensions of topographic organization that remain uncharacterized. Addressing this gap could clarify how APOE ε4 gene dose constrains the spatial substrate on which amyloid‐targeting therapies act and inform the design of genotype‐stratified clinical trials.

In this study, we conducted topographical analyses of amyloid‐PET data from two independent cohorts spanning cognitively unimpaired (CU), mild cognitive impairment (MCI), and dementia stages to quantify individual similarity to canonical AD amyloid maps and within‐group spatial homogeneity across APOE ε4 genotypes. We hypothesized that APOE ε4 carriers, particularly homozygotes, would exhibit a more biologically constrained pattern of amyloid accumulation, reflected by greater alignment with canonical AD topographies and higher within‐group spatial consistency compared with non‐carriers.

**RESEARCH IN CONTEXT**

1. **Systematic review of the literature**: We searched the literature on APOE ε4 and amyloid‐PET, identifying studies examining genotype‐related differences in amyloid burden and regional distribution. Prior work established that APOE ε4 increases global burden and shifts deposition toward temporoparietal hubs, but no study has quantified individual‐level topographic similarity to canonical AD maps or within‐genotype spatial consistency.
2. **Interpretation**: APOE ε4 gene dose constrains the spatial predictability of amyloid accumulation. Carriers, in particular homozygotes, show greater alignment with canonical AD topographies and higher inter‐individual homogeneity than non‐carriers, independent of global burden, revealing topographic stereotypy as a novel dimension of APOE ε4‐related amyloid pathology.
3. **Future directions**: Longitudinal amyloid and tau PET studies should determine whether topographic stereotypy predicts amyloid accumulation rate and downstream tau propagation. Clinical trials of anti‐amyloid therapies should prospectively test whether APOE ε4 homozygosity predicts more uniform spatial treatment response, informing genotype‐stratified eligibility criteria.

## 2 METHODS

### 2.1 Participants

Data used in the preparation of this article were obtained from the Alzheimer's Disease Neuroimaging Initiative (ADNI) database (adni.loni.usc.edu). ADNI was launched in 2003 as a public‐private partnership, led by Principal Investigator Michael W. Weiner, MD, with the primary goal of developing biomarkers for the early detection and tracking of AD. Further details are available at www.adni‐info.org. We included amyloid‐positive individuals from ADNI spanning the CU, MCI, and dementia stages. Amyloid positivity was determined using amyloid‐PET scans with florbetapir ([^1^ ^8^F]‐AV‐45), applying a standardized uptake value ratio (SUVR) cutoff of 1.11 using the whole cerebellum as the reference region, as recommended by the authors of the original analysis.^14^ Demographic, genetic, and clinical data were obtained from the ADNI database. APOE genotyping was performed as part of the ADNI baseline protocol, and participants were categorized according to the number of ε4 alleles as non‐carriers (zero ε4 alleles), heterozygotes (one ε4 allele), or homozygotes (two ε4 alleles).

To validate our findings, we analyzed an independent sample of amyloid‐positive individuals with MCI or dementia from the Geneva Memory Center (GMC). Amyloid positivity in the GMC cohort was assessed visually by an expert in nuclear medicine (VG).

Ethical approval was obtained from the Institutional Review Boards of all participating ADNI institutions and the Cantonal Ethics Committee of Geneva for the GMC cohort (PB_2016‐01346 and 2020_00403).

### 2.2 Imaging acquisition and processing

#### 2.2.1 ADNI acquisition

PET imaging scans in the ADNI cohort used [^1^ ^8^F]florbetapir (AV‐45) tracer. Participants received an intravenous bolus injection of 370 MBq (10 mCi ± 10%) of AV‐45, followed by a 30‐min uptake period in a resting state. Image acquisition began 50 min after injection and consisted of a dynamic 3D scan comprising four consecutive 5‐min frames. Scans were performed using PET or PET/computed tomography (CT) systems with appropriate attenuation correction, via either transmission scans (PET) or low‐dose CT scans (PET/CT). Subjects were closely monitored and repositioned between frames to minimize motion artifacts. All images were reconstructed using scanner‐specific parameters, quality‐checked for artifacts, and uploaded to the Laboratory of Neuroimaging for centralized storage and analysis.

For this study, we downloaded the fully preprocessed Digital Imaging and Communications in Medicine (DICOM) set 4# (“Co‐reg, Avg, Std Img, and Vox Siz, Uniform Resolution”).^15^ These standardized images were harmonized to a uniform spatial resolution using scanner‐specific 3D Gaussian smoothing kernels developed by the University of Michigan team, based on Hoffman phantom scans performed at each acquisition site.^16^ The target resolution was determined by the lowest‐performing scanners within ADNI and set to 6 mm full width at half maximum to ensure consistency across systems.^17^ Resulting preprocessed PET DICOMs have a standardized voxel size and spatial resolution.

#### 2.2.2 GMC acquisition

The amyloid‐PET images collected in GMC were acquired using [^1^ ^8^F]flutemetamol tracers using a protocol previously described in detail.^18^ [^1^ ^8^F]‐flutemetamol images were acquired 90 min after injection of 150 MBq for 20 min and were obtained on Siemens Biograph and Biograph Vision scanners (Siemens, Washington, DC), reconstructed using a 3D ordered subset expectation maximization iterative reconstruction, corrected for randoms, dead time, normalization, scatter, attenuation, and sensitivity. An in‐house workflow based on Statistical Parametric Mapping (SPM12; Wellcome Department of Cognitive Neurology, London, UK) was used for PET image processing.^18^

In both cohorts, voxel‐wise SUVR images were vectorized for each subject after resampling to 2 mm isotropic resolution and normalized to the default Montreal Neurological Institute (MNI) template using SPM12. All spatial co‐registrations were visually inspected by an experienced rater (MP) to ensure accurate alignment and consistent spatial resolution across all PET images. Two canonical amyloid PET reference patterns were used: one derived from AD patients included in the Centiloid Project^19^ and a second derived from an independent cohort of amyloid‐positive cognitively impaired individuals from the GMC who did not have available APOE ε4 genotyping. Information about the cohorts used to generate the canonical maps is provided in Table S1 and in the original study.^19^ Mean SUVR images were generated using the Image Calculator function in SPM12 to represent canonical AD amyloid distributions and were processed following the same preprocessing pipeline described earlier.^18^

### 2.3 Statistical analysis

Regional differences in amyloid‐PET uptake among the three APOE ε4 groups were assessed using voxel‐wise analyses. Gray matter‐restricted SUVR images, obtained by applying a probabilistic gray matter mask from SPM and normalized to MNI space, were entered into analyses of variance (ANOVAs) followed by pairwise two‐sample *t* tests in SPM12 to compare the three APOE ε4 groups and each group pair, respectively. Age and sex were included as nuisance covariates, and the statistical threshold was set at α < 0.05 with a cluster extent threshold of >100 voxels.

For each participant, similarity to the canonical AD distribution was quantified by computing Pearson correlation coefficients between individual amyloid‐PET maps and each reference (i.e., Centiloid and GMC maps) using vectorized data restricted to the gray matter. Group differences in correlation coefficients across APOE ε4 groups were tested using linear regression models entering the correlation coefficient as dependent variable and APOE ε4 genotype as the primary predictor (i.e., three levels: homozygotes, heterozygotes, and non‐carriers), adjusting for age, sex, and global amyloid burden. Estimated marginal means (EMMs) and pairwise contrasts between APOE ε4 groups were performed when results reached significance (α < 0.05), using the emmeans package in R with Tukey's correction for multiple comparisons. Then we used spatial correlation analysis to evaluate the local similarity of amyloid distribution across groups. To this end, we extracted local amyloid patterns within clusters of 5 × 5 × 5 voxels and compared them to the corresponding pattern in the canonical map using correlation analysis (Pearson correlation). This approach is similar to that used in imaging studies to measure structural similarity^20^ or regional homogeneity in functional magnetic resonance imaging (fMRI) data.^21^ Maps of local amyloid similarity were then averaged within each APOE ε4 group to derive group‐level maps. From these correlation maps between APOE ε4 groups and canonical maps, we applied a winner‐take‐all approach to assign each voxel to the APOE ε4 group showing the highest similarity value. In parallel, we computed voxel‐wise difference maps (e.g., homozygotes vs non‐carriers and heterozygotes vs non‐carriers) derived from the same local‐correlation maps, allowing us to identify brain regions where one group exhibited stronger or weaker correspondence to the canonical pattern compared with another.

To explore within‐group similarity, we computed inter‐subject correlation matrices of amyloid‐PET patterns for each APOE ε4 group. Specifically, we applied the same correlation approach as above (Pearson correlation) on the amyloid‐PET maps of each pair of subjects, using vectorized data restricted to the gray matter. From these matrices, all unique subject pairs – n(n−1)/2 – were extracted and entered in a linear regression model to test for APOE ε4‐related differences in similarity. Correlation values were entered as the dependent variable, APOE ε4 group as predictor, and age, sex, and global amyloid burden as covariates. Since each matrix element corresponds to a pair of subjects, it is not possible to assign individual covariates; therefore, we computed combined covariates based on the mean age, sex, and Centiloid values of each matrix pair. This analysis follows the inter‐subject similarity approach used in fMRI studies, where dyads are the unit of analysis and pair‐level covariates are used to explain inter‐subject similarity.^22^ In case of significance, we applied the same methodology described earlier, using EMM with Tukey correction for post hoc comparisons. The degree of spatial homogeneity of amyloid‐PET patterns within each APOE ε4 and clinical stage groups was also evaluated voxel‐wise by the computation of standard deviation (SD) maps. For each group, we extracted gray‐matter‐masked PET values for all subjects and, at each voxel, calculated the SD across subjects. These metrics quantify the intersubject variability of the amyloid signal at the voxel level: lower values indicate greater homogeneity (i.e., subjects within the group show more similar regional uptake), whereas higher values indicate greater heterogeneity. The same winner‐take‐all approach was applied to SD maps to identify, for each voxel, the APOE ε4 group with the highest homogeneity.

Analyses were conducted in the overall cohort and stratified by clinical stage within both the ADNI and GMC cohorts. However, due to smaller subgroup sizes in the GMC validation cohort, statistical comparisons were limited to APOE ε4 carriers (with one or two alleles) versus non‐carriers. All statistical analyses and visualization were performed in MATLAB R2025a, R (version 4.2.3), and MRIcroGL (Python API).

## 3 RESULTS

### 3.1 Participants

A total of 676 amyloid‐positive participants from the ADNI cohort were included, spanning CU (*n* = 160), MCI (*n* = 331), and dementia (*n* = 185) stages. The mean age of ADNI participants was 74 years (SD = 7), and 49% were female. Regarding APOE ε4, carriers represented 63% of the sample, including 49% heterozygotes (*n* = 329) and 14% homozygotes (*n* = 98). When comparing APOE ε4 groups, significant differences emerged in age (*p* < 0.001), MMSE scores (*p* < 0.001), AV45 SUVR (*p* < 0.001), and hippocampal volume (*p* = 0.010). Homozygotes were younger, were more cognitively impaired, showed the highest amyloid burden, and had the smallest hippocampal volumes. Clinical stage distribution also differed across groups (*p* < 0.001), with homozygotes presenting fewer CU individuals and more dementia cases than non‐carriers and heterozygotes. Demographic and clinical characteristics are summarized in Table 1 by APOE ε4 and in Table S2 stratified by clinical stage.

**TABLE 1:** Demographic, cognitive, and imaging characteristics across APOE ε4 genotype groups in ADNI cohort.

Variables	All *N* = 676	Non‐carriers *N* = 249	Heterozygotes *N* = 329	Homozygotes *N* = 98	*p* value
Age (years)	74 (7)	77 (7)^a^	74 (7)^b^	71 (8)^c^	<0.001
Sex (female)	329 (49%)	122 (49%)	166 (50%)	41 (42%)	0.3
Education (years)	16 (3)	16 (3)	16 (3)	16 (3)	0.8
MMSE	26.5 (3.3)	27.1 (2.9)^b^	26.2 (3.6)^a^	25.7 (3.1)^a^	<0.001
Clinical groups					<0.001
CU	160 (24%)	85 (34%)	68 (21%)	7 (7.1%)	
MCI	331 (49%)	116 (47%)	165 (50%)	50 (51%)	
Dementia	185 (27%)	48 (19%)	96 (29%)	41 (42%)	
AV45 SUVR	1.39 (0.18)	1.35 (0.18)^b^	1.40 (0.18)^a^	1.42 (0.15)^a^	<0.001
Hippocampal volume	6655 (1111)	6830 (1084)^b^	6570 (1123)^a^	6479 (1091)^a^	0.010

*Note*: Data are shown as mean (standard deviation) for continuous variables and as number of observations (percentage) for categorical variables. *p* values were derived from one‐way ANOVA for continuous variables and Fisher exact test for categorical variables. Post hoc pairwise comparisons were performed using independent *t* tests for continuous variables with Holm correction for multiple comparisons. Superscript letters correspond to the results of the post hoc comparison, namely, a  >  b  >  c.

Abbreviations: CU, cognitively unimpaired; MCI, mild cognitive impairment; MMSE, Mini‐Mental State Examination; SUVR, standardized uptake value ratio.

An independent validation cohort comprised 105 amyloid‐positive individuals with MCI (*n* = 86) and dementia (*n* = 19) from the GMC. In this cohort, 59% were APOE ε4 carriers (*n* = 62; heterozygotes *n* = 50, homozygotes *n* = 12), and 41% were non‐carriers (*n* = 43). A summary of demographic characteristics and amyloid burden across APOE ε4 groups in the GMC cohort is provided in Table S3.

### 3.2 Topography of amyloid across APOE ε4 groups

ANOVA analyses revealed widespread regional differences in amyloid uptake across APOE ε4 genotype groups (Figure 1), with prominent effects in lateral and medial frontal cortices, lateral temporal regions, precuneus, posterior cingulate cortex, and striatum. Pairwise voxel‐wise *t* tests showed that both APOE ε4 homozygotes and heterozygotes exhibited significantly higher amyloid deposition than non‐carriers across these regions, with the most extensive and robust effects observed in heterozygotes. Analyses conducted within each clinical stage showed similar effects in MCI, while in CU the effect was present only in heterozygotes. No detectable differences were observed in the dementia group (Supplementary Results and Figure S1). In the GMC validation cohort, voxel‐wise *t* tests comparing APOE ε4 carriers and non‐carriers showed higher amyloid burden in carriers, predominantly in the orbitofrontal and medial frontal cortices, as well as scattered neocortical regions (Figure S2).

**FIGURE 1:**
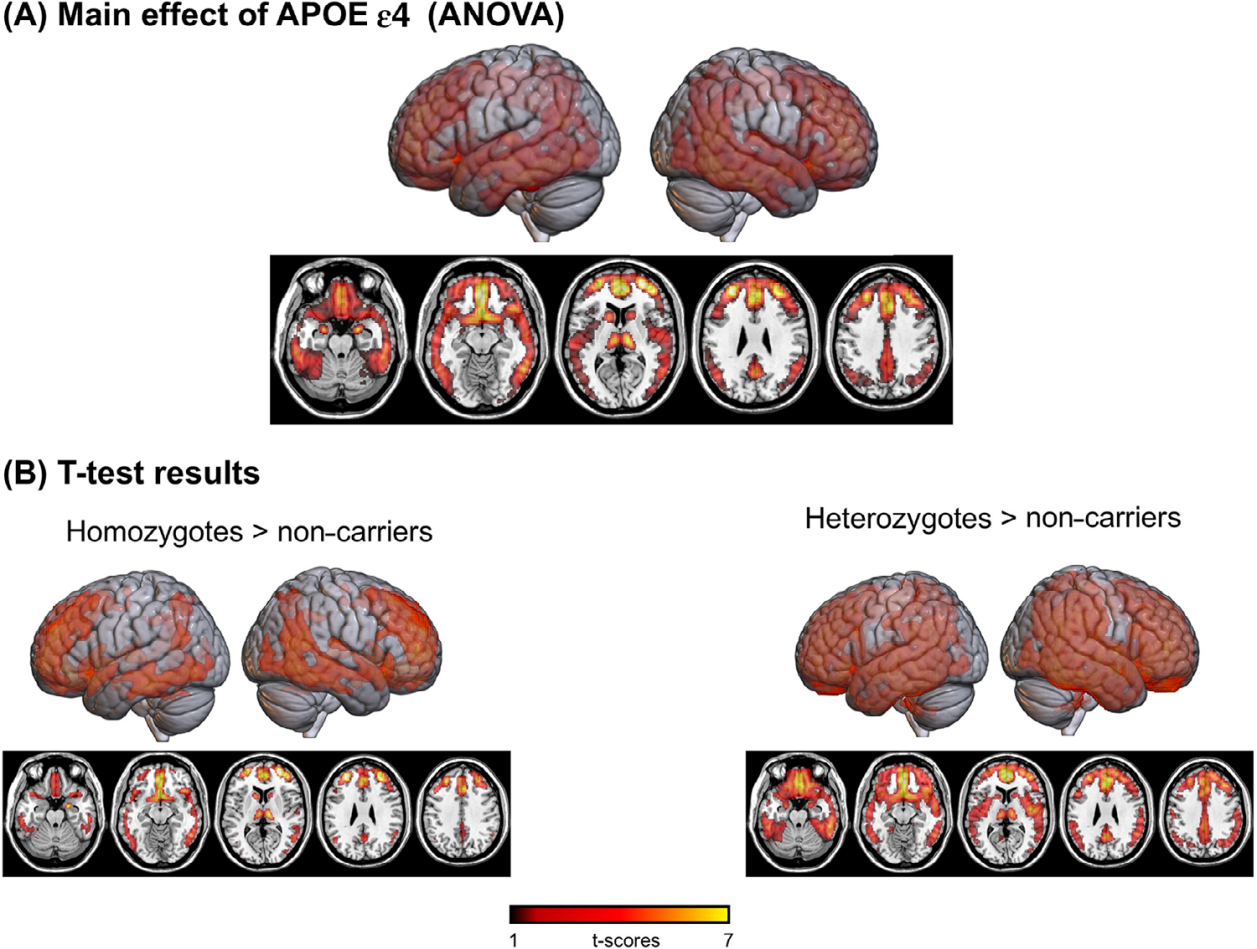
Voxel‐wise group differences in amyloid‐PET uptake across APOE ε4 genotypes. The significance clusters were based on α < 0.05 with a cluster‐extent threshold of >100 voxels and represented increased amyloid uptake in red–yellow (higher *t* values) for significant group differences.

### 3.3 Similarity to canonical Alzheimer's disease amyloid maps

APOE ε4 genotype was a strong predictor of amyloid‐PET pattern similarity for both canonical amyloid maps. In ADNI, for the canonical Centiloid map, APOE ε4 explained a significant proportion of variance in similarity scores (*F*[2,669] = 52.8, *p* < 0.001), with post hoc contrasts indicating significantly lower similarity in non‐carriers compared with both APOE ε4 heterozygotes (β = −0.03, SE = 0.01, *t*[669] = −4.92, *p* < 0.001) and homozygotes (β = −0.05, SE = 0.01, *t*[669] = −5.46, *p* < 0.001). The difference between heterozygotes and homozygotes showed a trend toward statistical significance (*p* = 0.07) (Figure 2). A similar pattern was observed for the GMC canonical map, where APOE ε4 genotype was again a significant predictor (*F*[2,669] = 27.78, *p* < 0.001) (Figure S3). Pairwise comparisons revealed that non‐carriers displayed lower similarity than heterozygotes (β = −0.024, SE = 0.006, *t*[669] = −4.09, *p* = 0.001) and homozygotes (β = −0.035, SE = 0.009, *t*[669] = −4.09, *p* = 0.001), while similarity did not differ significantly between heterozygotes and homozygotes (*p* = 0.37). For both canonical maps, age did not contribute significantly to similarity scores (*p* > 0.5), whereas sex and global amyloid load remained significant predictors (all *p* < 0.001), indicating that the APOE ε4 effect persists beyond overall amyloid burden. When analyses were stratified by clinical stage, APOE ε4 genotype showed a consistent association with amyloid‐PET pattern similarity, with the strongest effects observed in earlier disease stages, namely, CU and MCI (full description in Supplementary Results and Figure S4). When we relied on a winner‐take‐all approach to assign each voxel to the APOE ε4 group showing the highest similarity to the canonical maps, we confirmed the foregoing results and observed clearer group‐level effects (Figure 2). Full statistical outputs, including model summaries and all pairwise post hoc comparisons, as well as amyloid‐PET spatial maps, are provided in Tables S4 to S25 and Figures S5 to S7.

**FIGURE 2:**
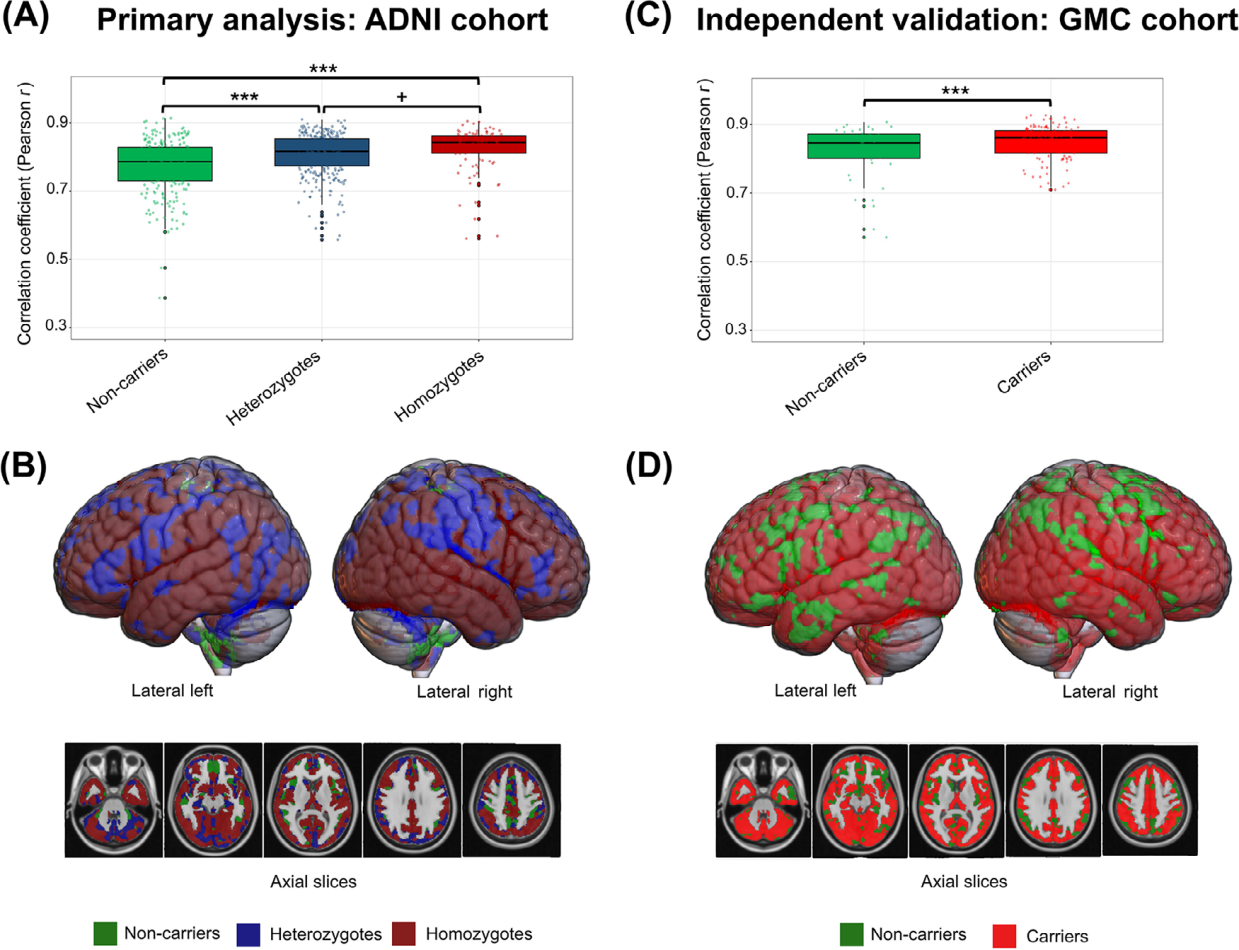
APOE ε4 carriership is associated with greater topographic similarity to the Centiloid canonical amyloid‐PET pattern across ADNI and GMC cohorts. (A and C) Boxplots of voxel‐wise correlation coefficients (Pearson *r*) quantifying the similarity between each subject's amyloid‐PET distribution and the canonical Centiloid amyloid map. In ADNI (A), similarity increases across APOE ε4 genotypes (non‐carriers, heterozygotes, homozygotes), whereas in the GMC validation cohort (C), carriers show significantly higher similarity than non‐carriers. (B) Voxelwise winner‐take‐all map for the ADNI cohort, displaying for each voxel the APOE ε4 group with the highest mean correlation to the canonical pattern. The widespread red (homozygotes), limited blue (heterozygotes), and minimal green (non‐carriers) indicate that topographic similarity to the canonical amyloid distribution is highest in homozygotes, intermediate in heterozygotes, and lowest in non‐carriers. (D) For the GMC cohort, winner‐take‐all maps are shown only for non‐carriers (green) and carriers (red), showing higher regional similarity to the canonical map in APOE ε4 carriers. ***p < 0.001; +*p* < 0.1.

Findings were further validated in the independent GMC cohort. In this cohort, APOE ε4 carriership was associated with higher similarity to the canonical Centiloid map (*F*[1,100] = 5.49, *p* = 0.021) and for the canonical GMC map (*F*[1,100] = 4.76, *p* = 0.031) (Figure 2). Across all models, global amyloid burden was a significant covariate, while age and sex showed smaller or inconsistent effects. In participants with dementia, the same models did not reveal a significant effect of APOE ε4 carriership (*p* > 0.05), whereas analyses conducted in the MCI group yielded results comparable to those observed in the whole sample (Figure S8).

### 3.4 Within‐group spatial similarity of amyloid deposition

In the ADNI whole cohort, APOE ε4 genotype was strongly associated with within‐group spatial similarity of amyloid‐PET patterns (*F*[2, 33,796] = 902.5, *p* < 0.001). Pairwise Tukey‐adjusted comparisons showed that heterozygotes exhibited higher spatial homogeneity than non‐carriers (β = −0.023, SE = 0.001, *t*[33,796] = −21.15, *p* < 0.001), and homozygotes showed even higher homogeneity than non‐carriers (β = −0.056, SE = 0.002, *t*[33,796] = −25.1, *p* < 0.001). Homozygotes also demonstrated higher spatial similarity than heterozygotes (β = −0.033, SE = 0.002, *t*[33,796] = −15.7, *p* < 0.001), indicating a clear dose‐dependent effect of APOE ε4 allele on inter‐subject amyloid pattern convergence (Figure 3). Across clinical stages, the APOE ε4 genotype continued to influence within‐group spatial homogeneity of amyloid‐PET patterns, although the magnitude and direction of effects varied across disease progression. In CU individuals, this was the only stage without a clear dose‐dependent increase in spatial homogeneity across APOE ε4 genotypes, a pattern likely influenced by the very small number of APOE ε4 homozygotes (*n* = 7) in this group. In contrast, both MCI and dementia showed a dose‐dependent effect, with spatial similarity progressively increasing from non‐carriers to heterozygotes and homozygotes (full description in Supplementary Results and Figure S9). Winner‐take‐all maps revealed that APOE ε4 homozygotes exhibited the greatest spatial homogeneity across widespread cortical and subcortical regions, whereas non‐carriers showed the highest homogeneity primarily in frontal and striatal areas (Figure 3). Model summaries with pairwise post hoc comparisons, and within‐group amyloid‐PET spatial maps are provided in Tables S26 to S36 and Figures S10 to S11.

**FIGURE 3:**
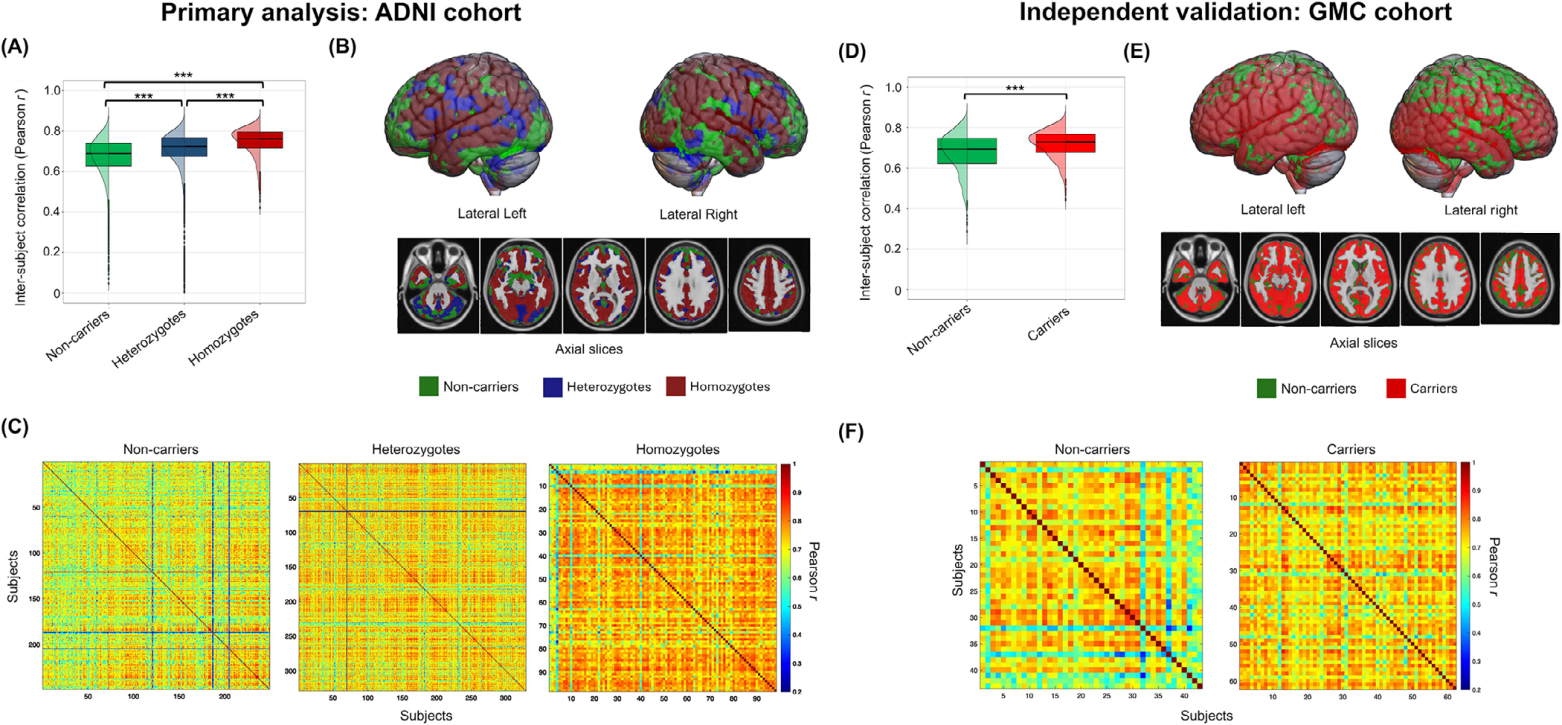
Within‐group similarity of amyloid‐PET topographies across APOE ε4 genotypes in ADNI and GMC cohorts. (A and D) Boxplots showing inter‐subject correlations (Pearson *r*) of amyloid‐PET signal within each APOE ε4 group in ADNI (A) and GMC (D) cohorts. Higher values indicate greater within‐group topographic similarity. (B and E) Voxelwise winner‐take‐all maps indicating, for each voxel, the APOE ε4 group with the highest within‐group consistency in amyloid‐PET topography. In the ADNI cohort (B), regional spatial homogeneity was greatest in APOE ε4 homozygotes (red), intermediate in heterozygotes (blue), and lowest in non‐carriers (green). In the GMC cohort (E), a similar pattern was observed, with higher spatial consistency in carriers (red) than in non‐carriers (green). (C and F) Inter‐subject correlation matrices in ADNI (C) and GMC (F), where cells represent the voxel‐wise Pearson correlation between two individuals’ amyloid‐PET maps. Warmer colors indicate higher spatial similarity, supporting greater homogeneity of amyloid deposition in APOE ε4 carriers, particularly homozygotes. ***p < 0.001.

Results were replicated in the independent GMC validation cohort, where APOE ε4 carrier status was strongly associated with spatial homogeneity (*F*[1,2189] = 140.3, *p* < 0.001), with carriers showing higher within‐group similarity compared with non‐carriers (Figure 3). A similar effect was observed among participants with MCI and dementia separately, where APOE ε4 carriership was significantly associated with greater homogeneity of amyloid deposition, with carriers again exhibiting higher within‐group correlations (Figure S12). Across all models, greater global amyloid burden and older age were consistently associated with increased within‐group similarity, while sex effects were minimal.

## 4 DISCUSSION

This study demonstrates that APOE ε4 shapes not only the magnitude but also the spatial predictability of amyloid deposition, in a gene–dose‐dependent manner. Beyond greater accumulation in AD‐vulnerable hubs, APOE ε4 carriers, and homozygotes in particular, exhibit amyloid patterns that are more tightly aligned with canonical AD topographies and more homogeneous across individuals, independent of overall amyloid burden. These effects were dose‐dependent, robust across PET tracers, and replicated across two independent cohorts.

The regions showing greater similarity and homogeneity among APOE ε4 carriers were localized in classical hubs of amyloid deposition, including the posterior cingulate cortex, precuneus, inferior parietal, and lateral temporal cortices, which are also the core regions used for Centiloid quantification.^19^ This spatial distribution aligns with prior studies showing that APOE ε4 carriers exhibit higher amyloid burden in these areas across disease stages. In CU individuals, APOE ε4 has been linked to early amyloid deposition in medial temporal and inferior parietal regions,^13^ while in mild to moderate AD, higher amyloid levels have been observed in temporoparietal regions,^23^ and in dementia, relatively lower frontal amyloid load has been reported among APOE ε4 carriers compared with non‐carriers.^24^, ^25^ Consistent with this literature, our findings revealed pronounced topographical differences mainly in the CU and MCI stages, suggesting a possible ceiling effect at the dementia stage where widespread amyloid deposition may mask APOE ε4‐related spatial distinctions.^11^, ^26^ While cortical saturation in dementia reduces the specificity of correlations with the canonical amyloid pattern, the persistence of high within‐group homogeneity in APOE ε4 carriers suggests that this saturation occurs in a highly synchronized and stereotyped manner. This is particularly relevant for APOE ε4 carriers, especially homozygotes, who not only exhibit higher overall amyloid burden but also tend to reach positivity thresholds much earlier, both in PET and cerebrospinal fluid measures,^11^, ^12^ and tend to plateau in amyloid accumulation sooner than non‐carriers.^27^ Indeed, amyloid positivity is two to three times more prevalent in APOE ε4 carriers compared with non‐carriers among CU individuals,^12^ and prior studies suggested that this group continued to accumulate amyloid even after surpassing the positivity threshold.^28^ Together, these observations support the idea that APOE ε4 shapes amyloid deposition both spatially and temporally, exerting its strongest effects in the early phases of the disease.

These findings provide topographic evidence in support of the probabilistic model of AD, demonstrating that APOE ε4 gene dose constrains the spatial patterning of amyloid accumulation, such that carriers, and homozygotes in particular, follow a less variable and more stereotyped trajectory. Whether individuals with ADAD would exhibit even greater topographic stereotypy than APOE ε4 homozygotes, as the probabilistic model predicts, remains an important hypothesis for future work.^4^ Nonetheless, conceptually these results align with the aforementioned model postulating a gradient of amyloid predictability driven by genetic determinants, in which amyloid precursor protein (APP), presenilin 1 (PSEN1), and PSEN2 mutations define a nearly fixed biological trajectory of pathology,^29^, ^30^ followed by APOE ε4 homozygotes and heterozygotes, and finally non‐carriers,^13^ whose amyloid accumulation may be more influenced by stochastic or environmental factors. This interpretation is biologically plausible because the genes responsible for autosomal dominant AD and APOE ε4 share amyloidogenic mechanisms. Mutations in APP, PSEN1, and PSEN2 promote early and widespread fibrillar amyloid deposition in highly stereotyped cortical networks,^29^ whereas APOE ε4 primarily promotes impaired clearance, enhanced aggregation, and increased seeding of amyloid.^31^ Despite acting through different molecular mechanisms (i.e., overproduction vs impaired clearance), both pathways converge on a common downstream phenotype of excessive fibrillar amyloid deposition that follows a remarkably conserved topographical pattern across genetic and sporadic forms of AD. In line with this, our observation of a gene–dose effect, with homozygotes showing greater amyloid spatial predictability than heterozygotes, further supports this gradient of biological determinism. This pattern is consistent with prior evidence showing that the frequency of amyloid positivity^6^, ^12^, ^32^ and the earlier onset of amyloid accumulation^7^, ^27^ both increase with the number of APOE ε4 alleles, reinforcing the idea that APOE ε4 dosage modulates not only the risk but also the spatial expression of amyloid pathology.

Our approach fits within a growing body of work that moves beyond regional amyloid burden to characterize the topographic organization of pathology at the individual level. Prior analyses applied similarity‐based methods in structural MRI and resting‐state fMRI to capture inter‐individual convergence of brain organization^33^, ^34^ and the spatial heterogeneity of tau and cortical atrophy using cluster‐based subtyping approaches.^35^ Building on conceptually similar approaches in neuroimaging, our work introduces spatial similarity and within‐group homogeneity of amyloid‐PET distributions as quantitative measures of pathological stereotypy, an aspect of AD biology that may serve as a sensitive readout for stratifying patients by the predictability of their disease trajectory.

Although this study advances our understanding of how APOE ε4 influences amyloid topography, several limitations should be considered when interpreting the findings. First, the cross‐sectional design prevents inference on the longitudinal pattern of amyloid pathology, a common constraint in AD studies using PET imaging.^9^ This limitation also extends to the disease timing, as the APOE ε4 gene dose reduces the age of onset of amyloidosis; therefore, chronological age, included as a covariate in our models, does not consistently serve as a proxy for time since amyloid onset. Although we stratified analyses by clinical stage to approximate disease progression, longitudinal investigations will be essential to establish whether the similarity and homogeneity measures identified here are detectable at the individual level. Second, the limited number of APOE ε4 homozygotes, particularly among CU individuals (*n* = 7), may have reduced statistical power to detect dose‐dependent effects in the earliest disease stages. Third, differences in amyloid tracers across datasets should also be acknowledged. ADNI participants were scanned with [^1^ ^8^F]florbetapir, whereas the validation cohort from GMC used [^1^ ^8^F]flutemetamol. Despite this methodological variability, the APOE ε4‐related effects on spatial similarity and homogeneity were replicated across cohorts, and both canonical reference maps, each derived from datasets using different tracers, produced consistent results. This cross‐tracer reproducibility strengthens the robustness and generalizability of our findings. Finally, future studies should incorporate longitudinal and multimodal imaging to determine whether the increased spatial homogeneity observed here predicts faster tau propagation or cognitive decline and whether it does so independently of the earlier onset of amyloidosis associated with APOE ε4 carriage. Such work will also help clarify whether the phenotypic convergence of amyloid patterns in APOE ε4 carriers extends to tau pathology and neurodegeneration. Recent longitudinal PET evidence indicates that APOE ε4 amplifies amyloid‐related tau accumulation, suggesting that its effects on tau spread are mostly mediated by amyloid pathology,^28^, ^36^, ^37^ and that this relationship is moderated by age and clinical stage.^11^ Conversely, other evidence points to a partially amyloid‐independent pathway, in which APOE ε4 may directly promote early tau deposition in initial Braak regions,^28^, ^38^, ^39^ while at later stages its effect on tau spread becomes increasingly dependent on amyloid pathology.^40^ These observations underscore the need to extend similarity and homogeneity analyses to longitudinal and tau‐PET data, to test whether the spatial convergence we identified in amyloid patterns is also present in tau and whether it reflects a modulatory role of APOE ε4 in the trajectory of the amyloid cascade.

Overall, the findings of this study contribute to the broader debate on the deterministic versus stochastic nature of AD pathology.^4^ Although APOE ε4 is the strongest common genetic risk factor for sporadic AD, it is not fully deterministic as many carriers remain CU even at advanced ages.^9^, ^10^ Nevertheless, the greater spatial homogeneity and stereotyped amyloid topographies observed in APOE ε4 carriers suggest that this allele also confers a more biologically constrained and predictable course of amyloid deposition compared with non‐carriers. This pattern supports the notion of a more deterministic progression of AD pathology in the early disease stages among APOE ε4 carriers, particularly homozygotes. These findings are directly related to the amyloid substrate that anti‐amyloid treatments target: A more homogeneous and canonically distributed amyloid accumulation reduces variability among individuals in the therapeutic target and reinforces the rationale for stratifying anti‐amyloid trials by APOE ε4 genotype. Whether this translates into differential treatment benefit remains unclear: Pooled analyses across anti‐amyloid trials have suggested a comparable or slightly more favorable effect in APOE ε4 carriers,^41^ whereas the pivotal trials showed little evidence of benefit in homozygotes, even without adjustment for baseline amyloid burden or clinical severity.^42^, ^43^ What our findings do show is that amyloid accumulates along a more predictable spatial course in APOE ε4 carriers, and particularly in homozygotes, a pattern that could be useful for understanding how amyloid spreads in AD.

## CONFLICTS OF INTEREST STATEMENT

GBF has received consulting fees through his institution from Biogen, Diadem, Roche, Eisai, Eli Lilly, Ac Immune, Novo Nordisk, Schwabe, Bromatech, AtonRâ, World Clinical Trials, and J&J Innovative Medicine. GBF has received payment or honoraria for lectures, presentations, speaker bureaus, manuscript writing, or educational events through his institution from Biogen, Roche, Novo Nordisk, GE HealthCare, and Vifor Pharma. VG received research support and speaker fees through her institution from GE Healthcare, Siemens Healthineers, Novo Nordisk, Janssen, and Novartis. All other authors declare no conflicts of interest. Author disclosures are available in the Supporting Information.

## CONSENT STATEMENT

In both cohorts, written informed consent was obtained from all participants or their legally authorized representatives at the time of enrollment, and all research was conducted in accordance with the Declaration of Helsinki.

## Supporting information



Supporting Information

Supporting Information
