# Supplementary material for: APOE ε4 gene dose drives a canonical and predictable topography of amyloid deposition
Source: Alzheimers Dement. 2026 Oct 29;22(10):e71935. doi: 10.1002/alz.71935 (PMC13719804; doi:10.1002/alz.71935)
Supplement: Supplementary file 2 [file ALZ-22-e71935-s002.docx]

**APOE4 gene dose drives a canonical and predictable topography of amyloid deposition**

Augusto J. Mendes; Cecilia Boccalini; Federica Ribaldi; Michela Pievani; Débora Peretti; Valentina Garibotto; Andrea Chincarini; Giovanni B. Frisoni

Index

Supplementary Table 1. Demographic characteristics and amyloid-PET burden in the cohort from the GMC without APOE4 genotyping.	2

Supplementary Table 2. Demographic, cognitive, and imaging characteristics across APOE4 genotype groups and clinical stages in ADNI cohort.	3

Supplementary Table 3. Demographic characteristics and amyloid-PET burden in the validation cohort from the GMC.	4

Supplementary Results	5

Supplementary Figure 1. Voxel-wise group differences in amyloid-PET uptake across APOE4 genotypes in cognitively unimpaired (CU) and mild cognitive impairment (MCI) participants.	8

Supplementary Figure 2. Voxel-wise comparison of amyloid-PET uptake between APOE4 carriers and non-carriers in the GMC validation cohort.	9

Supplementary Figure 3. APOE4 carriership is associated with greater topographic similarity to the GMC canonical amyloid PET pattern across ADNI and GMC cohorts.	10

Supplementary Figure 4. Similarity of APOE4 groups with canonical amyloid maps stratified by clinical group in ADNI cohort.	11

Supplementary Tables 4-9. Linear models and Tukey-adjusted pairwise comparisons for whole-cohort amyloid-PET spatial similarity with canonical maps.	12

Supplementary Tables 10-13. Linear models and Tukey-adjusted pairwise comparisons for amyloid-PET spatial similarity with canonical maps in CU participants.	14

Supplementary Tables 14-19. Linear models and Tukey-adjusted pairwise comparisons for amyloid-PET spatial similarity with canonical maps in MCI participants.	15

Supplementary Tables 20-25. Linear models and Tukey-adjusted pairwise comparisons for amyloid-PET spatial similarity with canonical maps in patients with dementia.	17

Supplementary Figure 5. Voxel-wise difference maps showing regional differences in similarity to canonical amyloid patterns between APOE4 homozygotes and non-carriers.	19

Supplementary Figure 6. Voxel-wise difference maps showing regional differences in similarity to canonical amyloid patterns between APOE4 heterozygotes and non-carriers.	20

Supplementary Figure 7. Voxel-wise difference maps showing regional differences in similarity to canonical amyloid patterns between APOE4 homozygotes and heterozygotes.	21

Supplementary Figure 8. Similarity of APOE4 groups with both canonical amyloid-PET maps in the GMC cohort.	22

Supplementary Figure 9. Within-group similarity of amyloid topographies across APOE4 groups and clinical stages in ADNI cohort.	23

Supplementary Figure 10. Spatial variability maps of amyloid-PET signal across APOE4 genotype groups and clinical stages.	24

Supplementary Figure 11. Voxel-wise group differences in spatial variability of amyloid-PET across APOE4 genotypes and clinical stages.	25

Supplementary Tables 26-28. Linear models and Tukey-adjusted pairwise comparisons for whole-cohort within-group spatial similarity.	26

Supplementary Tables 29-30. Linear models and Tukey-adjusted pairwise comparisons for within-group spatial similarity in CU participants.	27

Supplementary Tables 31-33. Linear models and Tukey-adjusted pairwise comparisons for within-group spatial similarity in MCI participants.	28

Supplementary Tables 34-36. Linear models and Tukey-adjusted pairwise comparisons for within-group spatial similarity in patients with dementia.	29

Supplementary Figure 12. Within-group spatial similarity of amyloid-PET topographies across APOE4 groups in the GMC validation cohort.	30

**Supplementary Table 1.** **Demographic characteristics and amyloid-PET burden in the cohort from the GMC without APOE4 genotyping.**

|  | **Overall**  N = 103 | **Dementia**  N = 54 | **MCI**  N = 49 | **p-value** |
| --- | --- | --- | --- | --- |
| Age (years) | 72 (7) | 71 (7) | 74 (6) | 0.026 |
| Sex |  |  |  | 0.3 |
| Female | 55 (53%) | 26 (48%) | 29 (59%) |  |
| Education (years) | 13.1 (4.1) | 12.9 (4.3) | 13.3 (3.9) | >0.9 |
| Amyloid-PET Centiloid | 81 (28) | 85 (28) | 77 (28) | 0.2 |

Data are shown as mean (standard deviation) for continuous variables and as number of observations (percentage) for categorical variables. P-values were derived from independent t-test for continuous variables and Fisher’s exact test for categorical variables.

Supplementary Table 2. Demographic, cognitive, and imaging characteristics across APOE4 genotype groups and clinical stages in ADNI cohort.

|  | **CU** | | | |  | **MCI** | | | |  | **Dementia** | | | |  |
| --- | --- | --- | --- | --- | --- | --- | --- | --- | --- | --- | --- | --- | --- | --- | --- |
|  | **-/-**  N = 85 | **e4/-**  N = 68 | **e4/e4**  N = 7 | **All**  N = 160 | **p-value** | **-/-**  N = 116 | **e4/-**  N = 165 | **e4/e4**  N = 50 | **All**  N = 331 | **p-value** | **-/-**  N = 48 | **e4/-**  N = 96 | **e4/e4**  N = 41 | **All**  N = 185 | **p-value** |
| Age (years) | 76.9 (6.1) | 73.7 (6.2) | 73.8 (11.2) | 75.4 (6.6) | 0.021 | 76.4 (7.5) | 73.5 (6.5) | 69.8 (6.8) | 74.0 (7.2) | <0.001 | 76.1 (8.6) | 75.0 (7.4) | 71.6 (7.9) | 74.5 (8.0) | 0.025 |
| Sex (female) | 52 (61%) | 46 (67%) | 4 (57%) | 102 (63%) | 0.7 | 49 (42%) | 72 (43%) | 23 (46%) | 144 (43%) | >0.9 | 21 (43%) | 48 (50%) | 14 (34%) | 83 (44%) | 0.2 |
| Education (years) | 16.3 (2.7) | 16.4 (2.7) | 17.9 (1.8) | 16.4 (2.7) | 0.14 | 15.7 (2.9) | 16.0 (2.8) | 16.1 (2.9) | 15.9 (2.8) | 0.6 | 15.6 (2.7) | 15.6 (2.8) | 15.8 (2.4) | 15.6 (2.7) | >0.9 |
| MMSE | 28.9 (1.2) | 28.9 (1.1) | 29.4 (0.5) | 28.9 (1.2) | 0.086 | 27.7 (1.8) | 27.5 (1.9) | 27.4 (2.1) | 27.6 (1.9) | 0.5 | 22.6 (2.4) | 22.1 (3.6) | 23.0 (1.9) | 22.4 (3.0) | 0.2 |
| AV45 SUVR | 1.28 (0.16) | 1.34 (0.17) | 1.36 (0.14) | 1.31 (0.17) | 0.10 | 1.36 (0.17) | 1.41 (0.18) | 1.41 (0.14) | 1.39 (0.17) | 0.04 | 1.45 (0.17) | 1.45 (0.17) | 1.46 (0.17) | 1.45 (0.17) | >0.9 |
| Hippocampal volume | 7277 (959) | 7316 (852) | 7368 (569) | 7298 (896) | >0.9 | 6870 (1016) | 6806 (942) | 6622 (1136) | 6800 (1000) | 0.5 | 5995 (965) | 5630 (956) | 6071 (948) | 5815 (971) | 0.041 |
| Data are shown as mean (standard deviation) for continuous variables and as number of observations (percentage) for categorical variables. P-values were derived from one-way ANOVA for continuous variables and Fisher’s exact test for categorical variables. | | | | | | | | | | | | | | | |
| CU: Cognitively Unimpaired; MCI: Mild Cognitive Impairment; -/-: Non-carriers; e4/-: Heterozygotes; e4/e4: Homozygotes. | | | | | | | | | | | | | | | |

**Supplementary Table 3.** **Demographic characteristics and amyloid-PET burden in the validation cohort from the GMC.**

|  | **Whole cohort** | | |  | **Dementia** | | |  | **MCI** | | |  |
| --- | --- | --- | --- | --- | --- | --- | --- | --- | --- | --- | --- | --- |
|  | **Carrier**  N = 62 | **Non-carrier**  N = 43 | **Overall**  N = 105 | **p-value** | **Carrier**  N = 11 | **Non-carrier**  N = 8 | **Overall**  N = 19 | **p-value** | **Carrier**  N = 51 | **Non-carrier**  N = 35 | **Overall**  N = 86 | **p-value** |
| Age (years) | 70.8 (6.4) | 72.8 (5.4) | 71.6 (6.0) | 0.088 | 72.5 (7.3) | 71.6 (7.7) | 72.1 (7.2) | 0.8 | 70.5 (6.2) | 73.1 (4.8) | 71.5 (5.8) | 0.031 |
| Sex (female) | 37 (60%) | 19 (44%) | 56 (53%) | 0.2 | 5 (46%) | 4 (50%) | 9 (47%) | >0.9 | 32 (63%) | 15 (43%) | 47 (55%) | 0.081 |
| Education (years) | 13.9 (3.9) | 13.8 (3.9) | 13.9 (3.9) | 0.8 | 12.4 (3.3) | 14.4 (3.0) | 13.2 (3.2) | 0.2 | 14.3 (4.0) | 13.6 (4.1) | 14.0 (4.0) | 0.5 |
| APOE4 carriership |  |  |  | <0.001 |  |  |  | <0.001 |  |  |  | <0.001 |
| Heterozygotes | 50 (81%) | 0 (0%) | 50 (47%) |  | 7 (64%) | 0 (0%) | 7 (37%) |  | 43 (84%) | 0 (0%) | 43 (50%) |  |
| Homozygotes | 12 (19%) | 0 (0%) | 12 (11%) |  | 4 (36%) | 0 (0%) | 4 (21%) |  | 8 (16%) | 0 (0%) | 8 (9%) |  |
| Non-carriers | 0 (0%) | 43 (100%) | 43 (41%) |  | 0 (0%) | 8 (100%) | 8 (42%) |  | 0 (0%) | 35 (100%) | 35 (41%) |  |
| Amyloid-PET Centiloid | 80.3 (26.3) | 72.9 (30.8) | 77.3 (28.3) | 0.2 | 86.3 (24.6) | 80.9 (48.5) | 84.0 (35.5) | 0.8 | 79.0 (26.7) | 71.1 (25.9) | 75.8 (26.5) | 0.2 |
| Data are shown as mean (standard deviation) for continuous variables and as number of observations (percentage) for categorical variables. P-values were derived from independent t-test for continuous variables and Fisher’s exact test for categorical variables. | | | | | | | | | | | | |
|  | | | | | | | | | | | | |

**Supplementary Results**

**Topography of Amyloid across APOE4 Groups**

ANOVA results revealed widespread regional differences in amyloid load among the three APOE4 groups in CU and MCI participants (Supplementary Figure 1), but not in those with dementia. These differences were mainly localized to the lateral and medial frontal cortices, lateral temporal cortices, precuneus, posterior cingulate, and striatum. Voxel-wise t-tests indicated that both APOE4 homozygotes and heterozygotes exhibited significantly higher amyloid load than non-carriers in these regions, with effects being most pronounced in heterozygotes within the MCI group. In CU individuals, differences between heterozygotes and non-carriers remained significant, whereas no significant effects were observed in dementia.

**Similarity to Canonical Alzheimer’s Disease Amyloid Maps Stratified by Clinical Stage**

In CU participants, APOE4 genotype was significantly associated with the canonical amyloid-PET pattern, both for Centiloid (F(2,154) = 15.68, p < 0.001) and GMC (F(2,154) = 11.75, p < 0.001) maps (Supplementary Figure 4). Post-hoc analyses showed higher similarity in APOE4 heterozygotes compared with non-carriers (both p < 0.001), with no significant differences involving homozygotes. A similar pattern occurred at the MCI stage, where APOE4 genotype remained a strong predictor of similarity for both canonical maps (Centiloid: F(2,324) = 23.25, p < 0.001; GMC: F(2,324) = 11.97, p < 0.001). Post-hoc tests showed that non-carriers exhibited lower similarity than both heterozygotes (Centiloid: p = 0.012; GMC: p = 0.087) and homozygotes (Centiloid: p < 0.001; GMC: p = 0.011). Differences between heterozygotes and homozygotes were not significant for the GMC canonical map (p > 0.05), although a marginal increase in similarity among homozygotes compared with heterozygotes was observed for the Centiloid canonical map (p = 0.08). At the dementia stage, the effect of APOE4 genotype on similarity was attenuated, reaching significance only for the Centiloid map (F(2,179) = 3.57, p = 0.030), but not for the GMC map (p = 0.10). Post-hoc comparisons revealed only a marginal significant effect toward higher similarity in homozygotes compared with non-carriers for the Centiloid map (p = 0.09). In GMC cohort, APOE4 carriers who are MCI displayed higher similarity to Centiloid (F(1,132) = 12.1, p < 0.001) and GMC canonical map (F(1,132) = 7.82, p = 0.005) (Supplementary Figure 5). On the other hand, dementia APOE4 carriers have showed a marginal significant effect in the GMC canonical map (F(1,25) = 3.85, p = 0.06).

Winner-takes-all maps have showed similar results in each clinical group when compared to the whole cohort reported in the main manuscript. In CU, APOE4 heterozygotes showed the highest similarity to the canonical map in distributed cortical areas (frontal, parietal and temporal), while homozygotes showed higher similarity than the other APOE groups in local regions (lateral temporal cortex, fusiform and lingual areas, insular cortex). APOE4 homozygotes showed the highest similarity in almost all brain regions at the MCI stage, and in the majority of cortical and subcortical regions also at the dementia stage. However, at this stage the pattern was more heterogeneous, APOE4 non-carriers showing higher similarity in several cortical (medial and lateral parietal, medial occipital cortex) and subcortical (hippocampus) regions (Supplementary Figure 4). Similar topographical results were found in the GMC validation cohort in regards to the winner-takes-all maps (Supplementary Figure 8).

**Within-Group Spatial Similarity of Amyloid Deposition Stratified by Clinical Stage**

Analyses of within-group spatial similarity measured by inter-subject Pearson correlations revealed robust effects of APOE4 genotype across all clinical stages. Among CU participants, significant differences in homogeneity were observed according to APOE4 genotype (F(2,5863) = 307.0, p < 0.001). Heterozygotes showed higher within-group similarity than non-carriers (p < 0.001), while homozygotes exhibited lower homogeneity than heterozygotes (p < 0.001). At the MCI stage, the effect of APOE4 genotype in spatial homogeneity remained highly significant (F(2,21419) = 406.7, p < 0.001). Post-hoc analyses demonstrated a monotonic increase in spatial homogeneity from non-carriers to heterozygotes and homozygotes (all p < 0.001). In participants with dementia, APOE4 genotype continued to influence spatial homogeneity (F(2,6502) = 84.16, p < 0.001). Pairwise comparisons indicated a progressive increase in within-group spatial similarity with the number of APOE4 alleles, with heterozygotes showing greater homogeneity than non-carriers (p = 0.017) and homozygotes showing significantly higher values than non-carriers and heterozygotes (both p < 0.001) (Supplementary Figure 9). Same significant effects were replicated in GMC cohort both for MCI (F(1,1521) = 87.17, p < 0.001) and dementia (F(1,50) = 10.1, p = 0.002) (Supplementary Figure 12).

At the voxel-wise level, the winner-takes-all approach applied to SD maps confirmed that the highest homogeneity was observed in homozygotes at the MCI and dementia stages, while heterozygotes showed the highest homogeneity at the CU stage. High spatial homogeneity in MCI mapped to distributed cortical and subcortical regions and showed a similar but more localized pattern in dementia. At this stage, non-carriers showed the highest homogeneity in several frontal and striatal areas. In CU, the most homogeneous regions were assigned to heterozygotes, with only few unspecific areas assigned to homozygotes, probably due to the small size of this group (Supplementary Figure 9).

**Supplementary Figure 1. Voxel-wise group differences in amyloid-PET uptake across APOE4 genotypes in cognitively unimpaired (CU) and mild cognitive impairment (MCI) participants.** The significance clusters were based on α < 0.05 with a cluster-extent threshold of >100 voxels and represented increased amyloid uptake in red–yellow (higher t-values) for significant group differences.

**Supplementary Figure 2. Voxel-wise comparison of amyloid-PET uptake between APOE4 carriers and non-carriers in the GMC validation cohort.** The significance clusters were based on α < 0.05 with a cluster-extent threshold of >100 voxels and represented increased amyloid uptake in red–yellow (higher t-values) for significant group differences.

Carriers > Non-carriers

**Supplementary Figure 3. APOE4 carriership is associated with greater topographic similarity to the GMC canonical amyloid PET pattern across ADNI and GMC cohorts.** (A, C) Boxplots of voxelwise correlation coefficients (Pearson’s r) quantifying the similarity between each subject’s amyloid PET distribution and the canonical GMC amyloid map. In ADNI (A), similarity increases across APOE4 genotypes (non-carriers, heterozygotes, homozygotes), whereas in the GMC validation cohort (C), carriers show significantly higher similarity than non-carriers. (B) Voxelwise winner-take-all map for the ADNI cohort, displaying for each voxel the APOE4 group with the highest mean correlation to the canonical pattern. Results were similar to the ones presented in the main manuscript in regards to the Centiloid canonical map, namely, the widespread red (homozygotes), limited blue (heterozygotes), and minimal green (non-carriers) indicate that topographic similarity to the canonical amyloid distribution is highest in homozygotes, intermediate in heterozygotes, and lowest in non-carriers. (D) For the GMC cohort, winner-take-all maps are shown only for non-carriers (green) and carriers (red), showing higher regional similarity to the canonical map in APOE4 carriers. *** p < 0.001

**Supplementary Figure 4. Similarity of APOE4 groups with canonical amyloid maps stratified by clinical group in ADNI cohort.** (A, C) Boxplots of voxelwise correlations coefficients (Pearson’s r) between individuals from each APOE4 group with canonical amyloid topographies derived from the GMC cohort (panel A) and Centiloid dataset (panel C). Correlations are shown separately for CU, MCI, and dementia group. Higher values indicate stronger similarity to the canonical distribution. (B, D) Voxelwise winner-take-all maps for each clinical stage, indicating the APOE4 group with the highest mean correlation to the canonical GMC (panel B) and Centiloid (panel D) maps. These maps highlight regional predominance of canonical similarity patterns across APOE4 groups.

**Supplementary Tables 4-9. Linear models and Tukey-adjusted pairwise comparisons for whole-cohort amyloid-PET spatial similarity with canonical maps.** This table presents the linear model outputs and post-hoc pairwise contrasts evaluating the association between APOE4 genotype and whole-cohort similarity to the Centiloid and GMC canonical amyloid-PET maps. Models include age, sex, and global amyloid burden as covariates. Results correspond to the whole sample in ADNI and GMC cohort.

Whole ADNI cohort – Centiloid canonical map

|  | Df | Sum Sq | Mean Sq | F value | Pr(>F) | Sig. |
| --- | --- | --- | --- | --- | --- | --- |
| APOE4 | 2 | 0.5107 | 0.25535 | 52.7493 | < 2.2e-16 | *** |
| Age | 1 | 0.0004 | 0.00039 | 0.0813 | 0.775682 |  |
| Sex | 1 | 0.061 | 0.06097 | 12.5945 | 0.000414 | *** |
| AV45 | 1 | 1.758 | 1.75799 | 363.1583 | < 2.2e-16 | *** |
| Residuals | 669 | 3.2385 | 0.00484 |  |  |  |

| Pairwise APOE4 (Tukey-adjusted): | | |  |  |  |  |
| --- | --- | --- | --- | --- | --- | --- |
| contrast |  | estimate | SE | df | t.ratio | p.value |
| Non-carrier-Heterozygote | | -0.0294 | 0.00599 | 669 | -4.916 | <.0001 |
| Non-carrier-Homozygote | | -0.0474 | 0.00868 | 669 | -5.46 | <.0001 |
| Heterozygote-Homozygote | | -0.018 | 0.00813 | 669 | -2.21 | 0.0703 |

Whole ADNI cohort – GMC canonical map

|  | Df | Sum Sq | Mean Sq | F value | Pr(>F) | Sig. |
| --- | --- | --- | --- | --- | --- | --- |
| APOE4 | 2 | 0.2648 | 0.13238 | 27.7843 | 2.56E-12 | *** |
| Age | 1 | 0.0015 | 0.00146 | 0.3074 | 0.5795 |  |
| Sex | 1 | 0.0933 | 0.0933 | 19.5813 | 1.13E-05 | *** |
| AV45 | 1 | 0.6937 | 0.69372 | 145.6019 | < 2.2e-16 | *** |
| Residuals | 669 | 3.1875 | 0.00476 |  |  |  |

| Pairwise APOE4 (Tukey-adjusted): | | | |  | |  | |  | |  | |
| --- | --- | --- | --- | --- | --- | --- | --- | --- | --- | --- | --- |
| contrast |  | estimate | SE | | df | | t.ratio | | p.value | |  |
| Non-carrier-Heterozygote | | -0.0243 | 0.00594 | | 669 | | -4.09 | | 0.0001 | |  |
| Non-carrier-Homozygote | | -0.0352 | 0.00861 | | 669 | | -4.09 | | 0.0001 | |  |
| Heterozygote-Homozygote | | -0.0109 | 0.00807 | | 669 | | -1.355 | | 0.3656 | |  |

Whole GMC cohort – Centiloid canonical map

|  | Df | Sum Sq | Mean Sq | F value | Pr(>F) | Sig. |
| --- | --- | --- | --- | --- | --- | --- |
| APOE4 | 1 | 0.04153 | 0.04153 | 14.769 | 0.0001742 | *** |
| Age | 1 | 0.09185 | 0.09185 | 32.6657 | 5.13E-08 | *** |
| Sex | 1 | 0.01482 | 0.01482 | 5.2701 | 0.0229772 | * |
| Amyloid-PET Centiloid | 1 | 0.43675 | 0.43675 | 155.3337 | < 2.2e-16 | *** |
| Residuals | 162 | 0.4555 | 0.00281 |  |  |  |

Whole GMC cohort – GMC canonical map

|  | Df | Sum Sq | Mean Sq | F value | Pr(>F) | Sig. |
| --- | --- | --- | --- | --- | --- | --- |
| APOE4 | 1 | 0.02692 | 0.026916 | 11.3263 | 0.0009542 | *** |
| Age | 1 | 0.02355 | 0.023552 | 9.9107 | 0.0019566 | ** |
| Sex | 1 | 0.01409 | 0.014092 | 5.9301 | 0.015969 | * |
| Amyloid-PET Centiloid | 1 | 0.15693 | 0.156926 | 66.0351 | 1.08E-13 | *** |
| Residuals | 162 | 0.38498 | 0.002376 |  |  |  |

**Supplementary Tables 10-13. Linear models and Tukey-adjusted pairwise comparisons for amyloid-PET spatial similarity with canonical maps in CU participants.** This table presents the linear model outputs and post-hoc pairwise contrasts evaluating the association between APOE4 genotype and whole-cohort similarity to the Centiloid and GMC canonical amyloid-PET maps. Models include age, sex, and global amyloid burden as covariates. Results correspond to the CU participants in ADNI cohort.

CU from ADNI cohort – Centiloid canonical map

|  | Df | Sum Sq | Mean Sq | F value | Pr(>F) | Sig. |
| --- | --- | --- | --- | --- | --- | --- |
| APOE4 | 2 | 0.129 | 0.0645 | 15.6786 | 6.34E-07 | *** |
| Age | 1 | 0.03424 | 0.03424 | 8.3228 | 0.004477 | ** |
| Sex | 1 | 0.01494 | 0.01494 | 3.6326 | 0.05852 | . |
| AV45 | 1 | 0.3999 | 0.3999 | 97.2036 | < 2.2e-16 | *** |
| Residuals | 154 | 0.63356 | 0.00411 |  |  |  |

| Pairwise APOE4 (Tukey-adjusted): | | | |  | |  | |  | |  | |
| --- | --- | --- | --- | --- | --- | --- | --- | --- | --- | --- | --- |
| contrast |  | estimate | SE | | df | | t.ratio | | p.value | |  |
| Non-carrier-Heterozygote | | -0.0443 | 0.0109 | | 154 | | -4.065 | | 0.0002 | |  |
| Non-carrier-Homozygote | | -0.0101 | 0.0254 | | 154 | | -0.398 | | 0.9164 | |  |
| Heterozygote-Homozygote | | 0.0341 | 0.0255 | | 154 | | 1.338 | | 0.3762 | |  |

CU from ADNI cohort – GMC canonical map

|  | Df | Sum Sq | Mean Sq | F value | Pr(>F) | Sig. |
| --- | --- | --- | --- | --- | --- | --- |
| APOE4 | 2 | 0.08159 | 0.040797 | 11.7464 | 1.79E-05 | *** |
| Age | 1 | 0.04148 | 0.041477 | 11.9422 | 0.0007093 | *** |
| Sex | 1 | 0.03469 | 0.034694 | 9.9892 | 0.0018962 | ** |
| AV45 | 1 | 0.14315 | 0.143151 | 41.2162 | 1.60E-09 | *** |
| Residuals | 154 | 0.53487 | 0.003473 |  |  |  |

| Pairwise APOE4 (Tukey-adjusted): | | |  |  |  |  |
| --- | --- | --- | --- | --- | --- | --- |
| contrast |  | estimate | SE | df | t.ratio | p.value |
| Non-carrier-Heterozygote | | -0.04079 | 0.01 | 154 | -4.079 | 0.0002 |
| Non-carrier-Homozygote | | 0.00838 | 0.0234 | 154 | 0.359 | 0.9316 |
| Heterozygote-Homozygote | | 0.04918 | 0.0234 | 154 | 2.099 | 0.0933 |

**Supplementary Tables 14-19. Linear models and Tukey-adjusted pairwise comparisons for amyloid-PET spatial similarity with canonical maps in MCI participants.** This table presents the linear model outputs and post-hoc pairwise contrasts evaluating the association between APOE4 genotype and whole-cohort similarity to the Centiloid and GMC canonical amyloid-PET maps. Models include age, sex, and global amyloid burden as covariates. Results correspond to the MCI participants in ADNI and GMC cohort.

MCI from ADNI cohort – Centiloid canonical map

|  | Df | Sum Sq | Mean Sq | F value | Pr(>F) | Sig. |
| --- | --- | --- | --- | --- | --- | --- |
| APOE4 | 2 | 0.25261 | 0.12631 | 23.2487 | 3.67E-10 | *** |
| Age | 1 | 0.00376 | 0.00376 | 0.6924 | 0.405962 |  |
| Sex | 1 | 0.04327 | 0.04327 | 7.9647 | 0.005064 | ** |
| AV45 | 1 | 1.03628 | 1.03628 | 190.7446 | < 2.2e-16 | *** |
| Residuals | 324 | 1.76022 | 0.00543 |  |  |  |

| Pairwise APOE4 (Tukey-adjusted): | | | |  | |  | |  | |  | |
| --- | --- | --- | --- | --- | --- | --- | --- | --- | --- | --- | --- |
| contrast |  | estimate | SE | | df | | t.ratio | | p.value | |  |
| Non-carrier-Heterozygote | | -0.0264 | 0.00919 | | 324 | | -2.876 | | 0.0119 | |  |
| Non-carrier-Homozygote | | -0.0523 | 0.01311 | | 324 | | -3.985 | | 0.0002 | |  |
| Heterozygote-Homozygote | | -0.0258 | 0.01209 | | 324 | | -2.135 | | 0.0844 | |  |

MCI from ADNI cohort – GMC canonical map

|  | Df | Sum Sq | Mean Sq | F value | Pr(>F) | Sig. |
| --- | --- | --- | --- | --- | --- | --- |
| APOE4 | 2 | 0.13448 | 0.06724 | 11.973 | 9.62E-06 | *** |
| Age | 1 | 0.00281 | 0.00281 | 0.5006 | 0.47974 |  |
| Sex | 1 | 0.0684 | 0.0684 | 12.1799 | 0.00055 | *** |
| AV45 | 1 | 0.50439 | 0.50439 | 89.8127 | < 2.2e-16 | *** |
| Residuals | 324 | 1.81959 | 0.00562 |  |  |  |

| Pairwise APOE4 (Tukey-adjusted): | | | |  | |  | |  | |  | |
| --- | --- | --- | --- | --- | --- | --- | --- | --- | --- | --- | --- |
| contrast |  | estimate | SE | | df | | t.ratio | | p.value | |  |
| Non-carrier-Heterozygote | | -0.0198 | 0.00935 | | 324 | | -2.121 | | 0.0872 | |  |
| Non-carrier-Homozygote | | -0.0388 | 0.01333 | | 324 | | -2.91 | | 0.0108 | |  |
| Heterozygote-Homozygote | | -0.019 | 0.01229 | | 324 | | -1.544 | | 0.2717 | |  |

MCI from GMC cohort – Centiloid canonical map

|  | Df | Sum Sq | Mean Sq | F value | Pr(>F) | Sig. |
| --- | --- | --- | --- | --- | --- | --- |
| APOE4 | 1 | 0.03173 | 0.03173 | 12.0925 | 0.000686 | *** |
| Age | 1 | 0.07142 | 0.07142 | 27.2212 | 6.85E-07 | *** |
| Sex | 1 | 0.02437 | 0.02437 | 9.2866 | 0.002789 | ** |
| Amyloid-PET Centiloid | 1 | 0.37694 | 0.37694 | 143.6597 | < 2.2e-16 | *** |
| Residuals | 132 | 0.34635 | 0.00262 |  |  |  |

MCI from GMC cohort – GMC canonical map

|  | Df | Sum Sq | Mean Sq | F value | Pr(>F) | Sig. |
| --- | --- | --- | --- | --- | --- | --- |
| APOE4 | 1 | 0.016734 | 0.016734 | 7.8178 | 0.005946 | ** |
| Age | 1 | 0.011827 | 0.011827 | 5.5251 | 0.020227 | * |
| Sex | 1 | 0.020733 | 0.020733 | 9.6861 | 0.002277 | ** |
| Amyloid-PET Centiloid | 1 | 0.151194 | 0.151194 | 70.6332 | 6.01E-14 | *** |
| Residuals | 132 | 0.282552 | 0.002141 |  |  |  |

**Supplementary Tables 20-25. Linear models and Tukey-adjusted pairwise comparisons for amyloid-PET spatial similarity with canonical maps in patients with dementia.** This table presents the linear model outputs and post-hoc pairwise contrasts evaluating the association between APOE4 genotype and whole-cohort similarity to the Centiloid and GMC canonical amyloid-PET maps. Models include age, sex, and global amyloid burden as covariates. Results correspond to patients with dementia in ADNI and GMC cohort.

Patients with dementia from ADNI cohort – Centiloid canonical map

|  | Df | Sum Sq | Mean Sq | F value | Pr(>F) | Sig. |
| --- | --- | --- | --- | --- | --- | --- |
| APOE4 | 2 | 0.02567 | 0.012837 | 3.5746 | 0.0300443 | * |
| Age | 1 | 0.04463 | 0.044633 | 12.4289 | 0.0005369 | *** |
| Sex | 1 | 0.00788 | 0.00788 | 2.1942 | 0.1402867 |  |
| AV45 | 1 | 0.15943 | 0.159427 | 44.3955 | 3.20E-10 | *** |
| Residuals | 179 | 0.6428 | 0.003591 |  |  |  |

| Pairwise APOE4 (Tukey-adjusted): | | |  |  |  |  |
| --- | --- | --- | --- | --- | --- | --- |
| contrast |  | estimate | SE | df | t.ratio | p.value |
| Non-carrier-Heterozygote | | -0.00753 | 0.0106 | 179 | -0.707 | 0.7596 |
| Non-carrier-Homozygote | | -0.02774 | 0.0131 | 179 | -2.114 | 0.0898 |
| Heterozygote-Homozygote | | -0.02021 | 0.0115 | 179 | -1.763 | 0.1851 |

Patients with dementia from ADNI cohort – GMC canonical map

|  | Df | Sum Sq | Mean Sq | F value | Pr(>F) | Sig. |
| --- | --- | --- | --- | --- | --- | --- |
| APOE4 | 2 | 0.0168 | 0.008402 | 2.313 | 0.101914 |  |
| Age | 1 | 0.01421 | 0.014214 | 3.9129 | 0.049452 | * |
| Sex | 1 | 0.00225 | 0.002252 | 0.62 | 0.432107 |  |
| AV45 | 1 | 0.03592 | 0.035916 | 9.887 | 0.001949 | ** |
| Residuals | 179 | 0.65025 | 0.003633 |  |  |  |

| Pairwise APOE4 (Tukey-adjusted): | | |  |  |  |  |
| --- | --- | --- | --- | --- | --- | --- |
| contrast |  | estimate | SE | df | t.ratio | p.value |
| Non-carrier-Heterozygote | | -0.00809 | 0.0107 | 179 | -0.755 | 0.7311 |
| Non-carrier-Homozygote | | -0.02389 | 0.0132 | 179 | -1.81 | 0.1691 |
| Heterozygote-Homozygote | | -0.01581 | 0.0115 | 179 | -1.371 | 0.3583 |

Patients with dementia from GMC cohort – Centiloid canonical map

|  | Df | Sum Sq | Mean Sq | F value | Pr(>F) | Sig. |
| --- | --- | --- | --- | --- | --- | --- |
| APOE4 | 1 | 0.009922 | 0.009922 | 2.7284 | 0.1110873 |  |
| Age | 1 | 0.021932 | 0.021932 | 6.0309 | 0.0213543 | * |
| Sex | 1 | 0.001154 | 0.001154 | 0.3175 | 0.5781608 |  |
| Amyloid-PET Centiloid | 1 | 0.065442 | 0.065442 | 17.9956 | 0.0002655 | *** |
| Residuals | 25 | 0.090914 | 0.003637 |  |  |  |

Patients with dementia from GMC cohort – GMC canonical map

|  | Df | Sum Sq | Mean Sq | F value | Pr(>F) | Sig. |
| --- | --- | --- | --- | --- | --- | --- |
| APOE4 | 1 | 0.01213 | 0.0121302 | 3.8465 | 0.06108 | . |
| Age | 1 | 0.017084 | 0.0170844 | 5.4175 | 0.02833 | * |
| Sex | 1 | 0.000417 | 0.0004169 | 0.1322 | 0.7192 |  |
| Amyloid-PET Centiloid | 1 | 0.014949 | 0.014949 | 4.7404 | 0.03911 | * |
| Residuals | 25 | 0.078839 | 0.0031535 |  |  |  |

**Supplementary Figure 5. Voxel-wise difference maps showing regional differences in similarity to canonical amyloid patterns between APOE4 homozygotes and non-carriers.** For each clinical stage (All, CU, MCI, Dementia), voxel-wise maps display areas where the local spatial correlation between APOE4 group’s amyloid-PET images and either the GMC canonical map (left panel) or the Centiloid canonical map (right panel) differed. Red regions indicate voxels where APOE4 homozygotes show higher local correlation with the canonical amyloid map relative to non-carriers, whereas green regions indicate higher correlation in non-carriers compared with homozygotes. **Supplementary Figure 6. Voxel-wise difference maps showing regional differences in similarity to canonical amyloid patterns between APOE4 heterozygotes and non-carriers.** For each clinical stage (All, CU, MCI, Dementia), voxel-wise maps display areas where the local spatial correlation between APOE4 group’s amyloid-PET images and either the GMC canonical map (left panel) or the Centiloid canonical map (right panel) differed. Red regions indicate voxels where APOE4 heterozygotes show higher local correlation with the canonical amyloid map relative to non-carriers, whereas green regions indicate higher correlation in non-carriers compared with heterozygotes.

**Supplementary Figure 7. Voxel-wise difference maps showing regional differences in similarity to canonical amyloid patterns between APOE4 homozygotes and heterozygotes.** For each clinical stage (All, CU, MCI, Dementia), voxel-wise maps display areas where the local spatial correlation between APOE4 group’s amyloid-PET images and either the GMC canonical map (left panel) or the Centiloid canonical map (right panel) differed. Red regions indicate voxels where APOE4 homozygotes show higher local correlation with the canonical amyloid map relative to heterozygotes, whereas green regions indicate higher correlation in heterozygotes compared with homozygotes.

**Supplementary Figure 8. Similarity of APOE4 groups with canonical amyloid maps stratified by clinical group in GMC cohort.** (A, C) Boxplots of voxelwise correlations coefficients (Pearson’s r) between individuals from each APOE4 group with canonical amyloid topographies derived from the GMC cohort (panel A) and Centiloid dataset (panel C). Correlations are shown separately for MCI, dementia, and all. Higher values indicate stronger similarity to the canonical distribution. (B, D) Voxelwise winner-take-all maps for each clinical stage, indicating the APOE4 group with the highest mean correlation to the canonical GMC (panel B) and Centiloid (panel D) maps. These maps highlight regional predominance of canonical similarity patterns across APOE4 groups.

**Supplementary Figure 9.** **Within-group similarity of amyloid topographies across APOE4 groups and clinical stages in ADNI cohort.** (A) Boxplots of inter-subject correlations (Pearson’s r) within each APOE4 group (non-carriers, heterozygotes, homozygotes) for CU, MCI, and dementia stages. Higher values indicate greater spatial similarity of amyloid deposition patterns within the group. (B) Voxelwise winner-take-all maps depicting the APOE4 group with the highest within-group consistency of amyloid-PET topography at each voxel.

**Supplementary Figure 10. Spatial variability maps of amyloid-PET signal across APOE4 genotype groups and clinical stages.** Standard deviation (SD) maps illustrate the voxel-wise variability of amyloid deposition within each APOE4 group shown across the whole cohort and within CU, MCI, and dementia subgroups. Warm colors (yellow–red) indicate higher SD, reflecting greater inter-individual variability, whereas cool colors (blue–green) represent lower SD and thus higher spatial consistency of amyloid deposition within the group. For each clinical stage, the display range for dispersion values (SD) was determined using percentile thresholds (1st–99th percentile) computed across all APOE groups.

**Supplementary Figure 11. Voxel-wise group differences in spatial variability of amyloid-PET across APOE4 genotypes and clinical stages.** Difference maps show voxel-wise contrasts in within-group spatial variability (SD) of amyloid-PET signal between APOE4 genotype groups displayed for the whole cohort (All) and for CU, MCI, and dementia subgroups. Red regions indicate voxels where the first group in each comparison shows higher spatial consistency of amyloid deposition (lower SD), while green regions indicate voxels where the second group shows lower spatial consistency.

**Supplementary Tables 26-28. Linear models and Tukey-adjusted pairwise comparisons for whole-cohort within-group spatial similarity.** This table reports the linear model outputs and post-hoc pairwise contrasts evaluating the association between APOE4 genotype and within-group spatial similarity (inter-subject voxelwise correlations of amyloid-PET patterns). Models include age, sex, and global amyloid burden as covariates. Results are shown separately for the ADNI and GMC cohorts and correspond to the whole sample within each dataset.

Whole ADNI cohort

|  | Df | Sum Sq | Mean Sq | F value | Pr(>F) | Sig. |
| --- | --- | --- | --- | --- | --- | --- |
| APOE4 | 2 | 14.224 | 7.1118 | 902.491 | < 2.2e-16 | *** |
| Mean Age | 1 | 0.219 | 0.2189 | 27.777 | 1.37E-07 | *** |
| Sex Pair | 1 | 1.111 | 1.1107 | 140.947 | < 2.2e-16 | *** |
| Mean AV45 | 1 | 19.993 | 19.9929 | 2537.103 | < 2.2e-16 | *** |
| Residuals | 33796 | 266.319 | 0.0079 |  |  |  |

| Pairwise APOE4(Tukey-adjusted): | |  |  |  |  |
| --- | --- | --- | --- | --- | --- |
| contrast | estimate | SE | df | t.ratio | p.value |
| Non-carrier - Heterozygote | -0.0233 | 0.0011 | 33796 | -21.149 | <.0001 |
| Non-carrier - Homozygote | -0.0559 | 0.00223 | 33796 | -25.06 | <.0001 |
| Heterozygote - Homozygote | -0.0326 | 0.00208 | 33796 | -15.677 | <.0001 |

Whole GMC cohort

|  | Df | Sum Sq | Mean Sq | F value | Pr(>F) | Sig. |
| --- | --- | --- | --- | --- | --- | --- |
| APOE4 | 1 | 0.9689 | 0.96889 | 186.934 | < 2.2e-16 | *** |
| Mean Age | 1 | 0.3904 | 0.3904 | 75.3227 | < 2.2e-16 | *** |
| Sex Pair | 1 | 0.0362 | 0.03623 | 6.9908 | 0.008251 | ** |
| Mean Centiloid | 1 | 2.8391 | 2.83913 | 547.7722 | < 2.2e-16 | *** |
| Residuals | 2189 | 11.3457 | 0.00518 |  |  |  |

**Supplementary Tables 29-30. Linear models and Tukey-adjusted pairwise comparisons for within-group spatial similarity in CU participants.** This table reports the linear model outputs and post-hoc pairwise contrasts evaluating the association between APOE4 genotype and within-group spatial similarity (inter-subject voxelwise correlations of amyloid-PET patterns). Models include age, sex, and global amyloid burden as covariates. Results are shown for the CU participants from ADNI cohorts.

CU from ADNI

|  | Df | Sum Sq | Mean Sq | F value | Pr(>F) | Sig. |
| --- | --- | --- | --- | --- | --- | --- |
| APOE4 | 2 | 3.467 | 1.7334 | 307.0448 | < 2.2e-16 | *** |
| Mean Age | 1 | 0.721 | 0.72144 | 127.7918 | < 2.2e-16 | *** |
| Sex Pair | 1 | 0.003 | 0.00289 | 0.5123 | 0.4742 |  |
| Mean AV45 | 1 | 0.167 | 0.16747 | 29.6648 | 5.35E-08 | *** |
| Residuals | 5863 | 33.099 | 0.00565 |  |  |  |

| Pairwise APOEGroup (Tukey-adjusted): | |  |  |  |  |
| --- | --- | --- | --- | --- | --- |
| contrast | estimate | SE | df | t.ratio | p.value |
| Non-carrier - Heterozygote | -0.0541 | 0.0022 | 5863 | -24.649 | <.0001 |
| Non-carrier - Homozygote | 0.0111 | 0.0165 | 5863 | 0.672 | 0.7797 |
| Heterozygote - Homozygote | 0.0652 | 0.0165 | 5863 | 3.956 | 0.0002 |

**Supplementary Tables 31-33. Linear models and Tukey-adjusted pairwise comparisons for within-group spatial similarity in MCI participants.** This table reports the linear model outputs and post-hoc pairwise contrasts evaluating the association between APOE4 genotype and within-group spatial similarity (inter-subject voxelwise correlations of amyloid-PET patterns). Models include age, sex, and global amyloid burden as covariates. Results are shown separately for the ADNI and GMC cohorts and correspond to the MCI participants within each dataset.

MCI participants from ADNI

|  | Df | Sum Sq | Mean Sq | F value | Pr(>F) | Sig. |
| --- | --- | --- | --- | --- | --- | --- |
| APOE4 | 2 | 7.452 | 3.7261 | 406.729 | < 2.2e-16 | *** |
| Mean Age | 1 | 0.318 | 0.3183 | 34.741 | 3.82E-09 | *** |
| Sex Pair | 1 | 1.315 | 1.3148 | 143.521 | < 2.2e-16 | *** |
| Mean AV45 | 1 | 18.217 | 18.2171 | 1988.507 | < 2.2e-16 | *** |
| Residuals | 21419 | 196.224 | 0.0092 |  |  |  |

| Pairwise APOE4 (Tukey-adjusted): | |  |  |  |  |
| --- | --- | --- | --- | --- | --- |
| contrast | estimate | SE | df | t.ratio | p.value |
| Non-carrier - Heterozygote | -0.0191 | 0.00151 | 21419 | -12.643 | <.0001 |
| Non-carrier - Homozygote | -0.0545 | 0.00312 | 21419 | -17.473 | <.0001 |
| Heterozygote - Homozygote | -0.0354 | 0.0029 | 21419 | -12.208 | <.0001 |

MCI participants from GMC

|  | Df | Sum Sq | Mean Sq | F value | Pr(>F) | Sig. |
| --- | --- | --- | --- | --- | --- | --- |
| APOE4 | 1 | 0.4072 | 0.4072 | 87.1736 | < 2.2e-16 | *** |
| Mean Age | 1 | 0.1545 | 0.15449 | 33.0731 | 1.07E-08 | *** |
| Sex Pair | 1 | 0.0139 | 0.0139 | 2.9747 | 0.08478 | . |
| Mean Centiloid | 1 | 2.0518 | 2.05177 | 439.2444 | < 2.2e-16 | *** |
| Residuals | 1521 | 7.1048 | 0.00467 |  |  |  |

**Supplementary Tables 34-36. Linear models and Tukey-adjusted pairwise comparisons for within-group spatial similarity in patients with dementia.** This table reports the linear model outputs and post-hoc pairwise contrasts evaluating the association between APOE4 genotype and within-group spatial similarity (inter-subject voxelwise correlations of amyloid-PET patterns). Models include age, sex, and global amyloid burden as covariates. Results are shown separately for the ADNI and GMC cohorts and correspond to the patients with dementia within each dataset

Patients with dementia from ADNI

|  | Df | Sum Sq | Mean Sq | F value | Pr(>F) | Sig. |
| --- | --- | --- | --- | --- | --- | --- |
| APOE4 | 2 | 0.825 | 0.41247 | 84.164 | < 2.2e-16 | *** |
| Mean Age | 1 | 0.394 | 0.39424 | 80.445 | < 2.2e-16 | *** |
| Sex Pair | 1 | 0.12 | 0.11997 | 24.48 | 7.70E-07 | *** |
| Mean AV45 | 1 | 1.636 | 1.63607 | 333.838 | < 2.2e-16 | *** |
| Residuals | 6502 | 31.865 | 0.0049 |  |  |  |

| Pairwise APOEGroup (Tukey-adjusted): | |  |  |  |  |
| --- | --- | --- | --- | --- | --- |
| contrast | estimate | SE | df | t.ratio | p.value |
| Non-carrier - Heterozygote | -0.00642 | 0.00235 | 6502 | -2.738 | 0.0171 |
| Non-carrier - Homozygote | -0.03366 | 0.00331 | 6502 | -10.153 | <.0001 |
| Heterozygote - Homozygote | -0.02724 | 0.00271 | 6502 | -10.051 | <.0001 |

Patients with dementia from GMC

|  | Df | Sum Sq | Mean Sq | F value | Pr(>F) | Sig. |
| --- | --- | --- | --- | --- | --- | --- |
| APOE4 | 1 | 0.053807 | 0.053807 | 10.0984 | 0.0025451 | ** |
| MeanAge | 1 | 0.063712 | 0.063712 | 11.9573 | 0.0011209 | ** |
| SexMix | 1 | 0.001021 | 0.001021 | 0.1917 | 0.6634058 |  |
| MeanCentiloid | 1 | 0.074835 | 0.074835 | 14.0449 | 0.0004634 | *** |
| Residuals | 50 | 0.266414 | 0.005328 |  |  |  |

**Supplementary Figure 12. Within-group spatial similarity of amyloid-PET topographies across APOE4 groups in the GMC validation cohort.** (A) Boxplots show inter-subject spatial similarity (Pearson’s r) of amyloid-PET maps within each APOE4 group for MCI, dementia, and the combined sample. Higher values indicate greater spatial similarity of amyloid deposition patterns within the group. (B) Voxelwise winner-take-all maps depicting the APOE4 group with the highest within-group consistency of amyloid-PET topography at each voxel.
